# Host Cell Receptor and Herpes Simplex Virus 1 Glycoprotein B Are Determinants of Low Temperature Entry

**DOI:** 10.3390/v18020163

**Published:** 2026-01-27

**Authors:** Colleen M. Lynch, McKenna A. Hull, Anthony V. Nicola

**Affiliations:** Department of Veterinary Microbiology and Pathology, College of Veterinary Medicine, Washington State University, Pullman, WA 99164, USA; colleen.lynch@wsu.edu (C.M.L.); mckenna.hull@wsu.edu (M.A.H.)

**Keywords:** herpes simplex virus 1, low temperature entry, glycoprotein B, receptor

## Abstract

Herpes simplex virus 1 (HSV-1) entry is a complex interplay of viral and host factors. The mechanisms of its regulation remain undefined. HSV-1 entry occurs via multiple distinct and cell-type dependent pathways, further complicating study of this process. HSV-1 strains with atypical entry properties aid in the elucidation of entry determinants. HSV-1 strain ANG path exhibits entry in Vero cells at 4 °C, whereas wild-type strains do not. We investigated the determinants of low temperature entry by HSV-1 ANG path in several cell types. The receptor nectin-2 mediated 4 °C entry of HSV-1 ANG path into CHO-K1 cells, but the related receptor nectin-1 did not, suggesting that gD-binding receptors are a determinant of HSV-1 entry at low temperatures. In HaCaT cells, both HSV-1 ANG path and wild-type strain KOS entered at 4 °C, while HSV-1 chimera 27/III, which contains KOS strain gB in the ANG path virus background, did not. This suggests that gB functions as a determinant of low temperature entry of HSV-1. Together, the findings suggest that there are multiple determinants and mechanisms of HSV-1 low temperature entry and that the requirements differ by cell type.

## 1. Introduction

Herpes simplex virus 1 (HSV-1), a widespread human pathogen, replicates in epithelial cells during both initial infection and following reactivation. Host cell fusion and entry are incompletely understood, yet essential to replication, transmission, and pathogenesis. HSV-1 entry is a complex, multi-step process that occurs via both endocytic and non-endocytic pathways [1,2,3,4]. The first step, attachment, is mediated by viral envelope glycoproteins B and C (gB and gC) interacting with cell surface heparan sulfate [5,6,7]. In a subset of cell types including human epidermal keratinocytes, HSV-1 is then endocytosed and ultimately fuses with an endosomal membrane [4,8]. In Vero cells or neurons, HSV-1 fuses directly with the plasma membrane [1,2,3,4,9,10,11,12,13,14]. A combination of viral and host cell factors determines HSV-1 fusion and selection of entry pathway [9,15,16,17,18,19,20].

HSV-1 entry mechanisms are typically studied at 37 °C in cell culture, in accordance with normal human core body temperature. However, entry at lower temperatures is of interest as these conditions can reduce cellular functions, such as endocytosis and protein function [21,22,23,24,25], and may have significance in the dynamic environment of surface exposed epithelia. Notably, low temperatures are restrictive for membrane fluidity and fusion [26,27,28,29] and thus should inhibit HSV-1 entry. We selected 4 °C to test as a temperature low enough to reliably inhibit endocytosis [21,22,23,24] and membrane fusion by other viruses [27,30]. Investigating how HSV-1 fusion occurs at 4 °C will aid in identifying determinants of fusion that are relevant to entry at 37 °C.

HSV-1 strain ANG path [31] is a laboratory-derived strain which exhibits several interesting entry and fusion phenotypes. ANG path is highly neuroinvasive, associated with 100% mortality in intraperitoneally infected mice [31] and mortality at markedly lower infectious doses via footpad inoculation compared to the ANG strain [32]. This increased pathogenicity is attributed to the A84G point mutation in ANG path glycoprotein D (gD) [32]. HSV-1 ANG path is syncytial and its glycoproteins are hyperfusogenic in a cell–cell fusion assay, phenotypes which are attributed to mutations in gB [18,33,34,35,36]. HSV-1 strain ANG, the parent of ANG path, enters Vero cells at 4 °C. The wild-type strain KOS does not enter under these conditions [33], despite entering by direct fusion with the plasma membrane [3]. This suggests that strain ANG can overcome some of the inhibitory effects of low temperatures on entry. We theorize that strain ANG path has a similar low temperature entry phenotype, enters other cell types at low temperatures, and could be a valuable tool for studying entry under these conditions.

HSV-1 ANG path enters cell types using receptors and entry pathways that are often different from the wild-type virus [15,18]. ANG path utilizes the nectin-2 receptor more efficiently than KOS, but is less efficient at utilizing the nectin-1 receptor favored by wild-type strains [15]. HSV also typically enters CHO cells, a model cell type commonly used for in vitro studies, via the pH-dependent endocytic pathway [8]. ANG path enters CHO-nectin-1 cells via this pathway, but in CHO-nectin-2 cells ANG path enters via direct fusion with the plasma membrane [15]. Thus, HSV-1 ANG path can be used to evaluate the effects of different entry receptors and pathways on low temperature entry.

HSV-1 enters human keratinocyte cell lines and mouse keratinocytes at temperatures as low as 7 °C [23,37]. HSV entry in epithelial cells is thought to predominantly occur via a non-conventional, low pH-dependent, endocytic pathway [4]; however, at low temperatures, this pathway should be unavailable for HSV entry due to the inhibition of cellular functions. Direct fusion of HSV-1 with the plasma membrane is exhibited during entry into neurons and other cell types. In these cases, cellular endocytosis of the virus is not required [3,4,11]. HSV-1 entry by direct penetration was detected in keratinocytes at low temperatures [23,37]. The cellular and viral factors that facilitate fusion at low temperatures is an important knowledge gap.

Based on the ability of HSV-1 ANG to enter at low temperatures, and of strain ANG path to utilize diverse receptors and entry pathways in common laboratory cell types, we hypothesize that HSV strain ANG path exhibits unique entry phenotypes in the human keratinocyte cell-derived cell line, HaCaT. In this study, we demonstrate that ANG path enters HaCaT cells efficiently at low temperatures despite entering via the pH-dependent endocytic pathway and that the hyperfusogenic gB of ANG path is essential to this phenotype. These findings support the use of HSV strain ANG path as a tool for further investigating atypical entry in physiologically relevant keratinocytes. Understanding the determinants of the unique entry and fusion activities of HSV-1 strain ANG will help decipher the HSV fusion reaction and entry process.

## 2. Materials and Methods

### 2.1. Cells and Viruses

CHO-K1 cells (American Type Culture Collection (ATCC), Manassas, VA, USA) were propagated in Ham’s F12 nutrient mixture (Gibco/Life Technologies, Grand Island, NY, USA), supplemented with 10% fetal bovine serum (FBS) (Atlanta Biologicals, Atlanta, GA, USA) and 1X PSG. Vero cells (ATCC) and HaCaT cells were propagated in Dulbecco’s Modified Eagle’s Medium (Thermo Fisher Scientific, Waltham, MA, USA) supplemented with 10% FBS and 1X PSG. CHO-nectin-2 (M2A) cells [38] were propagated in Ham’s F12 nutrient mixture, supplemented with 10% FBS, 1X PSG, 500 µg/mL G418 (Sigma-Aldrich, St. Louis, MO, USA), and 150 μg/mL puromycin (Sigma-Aldrich, St. Louis, MO, USA) to maintain stable transfection of the human nectin-2 receptor.

HSV-1 strain KOS was obtained from Priscilla Schaffer, Harvard University. HSV-1 strains ANG path [31] and chimeric strain KBang [36] were obtained from Thomas Holland, Wayne State University. HSV-1 chimeric strain 27/III [35] was obtained from Dietrich Falke, University of Mainz. HSV KBang is a chimeric strain with ANG path gB in a KOS background, and HSV-1 27/III is a chimeric strain with KOS gB in an ANG path background. All viruses were propagated and titer determined on Vero cells.

### 2.2. Antibodies

For plaque assays, cells were probed using rabbit anti-HSV polyclonal antibody HR50 (Biosynth International, Louisville, KY, USA) at a 1:400 dilution or goat anti-herpes simplex virus 1/2 polyclonal antibody (BioRad, Hercules, CA, USA) at a 1:250 dilution. Horseradish peroxidase recombinant Protein A (Invitrogen, Rockford, IL, USA) was used to detect primary antibody.

### 2.3. Plaque Assay

Herpes simplex virus 1 was titrated by limiting dilution. At 18–24 h p.i., culture medium was removed, and cells were fixed with ice-cold methanol-acetone solution (2:1 ratio) for 20 min at −20 °C and air-dried. Virus titer was determined by immunoperoxidase staining.

### 2.4. Nectin-1 Transfection

In a 10 cm dish, CHO-K1 cells were transfected with the pBG38 plasmid encoding human nectin-1 [39]. Transfections were performed using Attractene transfection reagent (Qiagen, Venlo, The Netherlands). Then, 24 h post-transfection, cells were trypsinized and re-seeded into twenty-four well-plates for use in plaque assays.

### 2.5. Low Temperature Entry Plaque Assay

Serial dilutions of HSV-1 (1:5) were prepared in carbonate-free, serum-free medium supplemented with 20 mM HEPES and 0.2% bovine serum albumin. Twenty-four well plate cell cultures and virus dilutions were equilibrated to 4 °C, 15 °C, or 37 °C for 15 min. Virus was added to cells (approximately 80 PFU/well), and cultures were incubated at 4 °C, 15 °C, or 37 °C for 2 h. Cells were treated with room temperature sodium citrate buffer (pH 3.0) at 37 °C for 5 min (Vero, CHO-nectin-2, CHO-nectin-1) or 3.5 min (HaCaT) to inactivate attached virus that had not entered the cells. At 18–24 h post-infection at 37 °C, virus titers were measured by plaque assay.

### 2.6. Effect of Ammonium Chloride on HSV-1 Entry

Stock solution of ammonium chloride (1.5 M) was prepared in water and subsequently diluted in complete DMEM. HaCaT cells grown in 24-well cell culture plates were treated with complete DMEM supplemented with ammonium chloride at twice the target concentration for 1 h at 37 °C. The NH_4_Cl concentrations used are non-cytotoxic to all cell types tested [8,40]. Virus was added at 100 PFU/well in the continued presence of the agent for 6 h. Medium was removed and replaced with complete DMEM. At 18 hpi, viral entry and infectivity was measured via plaque assay.

## 3. Results

### 3.1. Nectin-2 Mediates 4 °C Entry of HSV-1 ANG Path into CHO Cells

**Figure 1:**
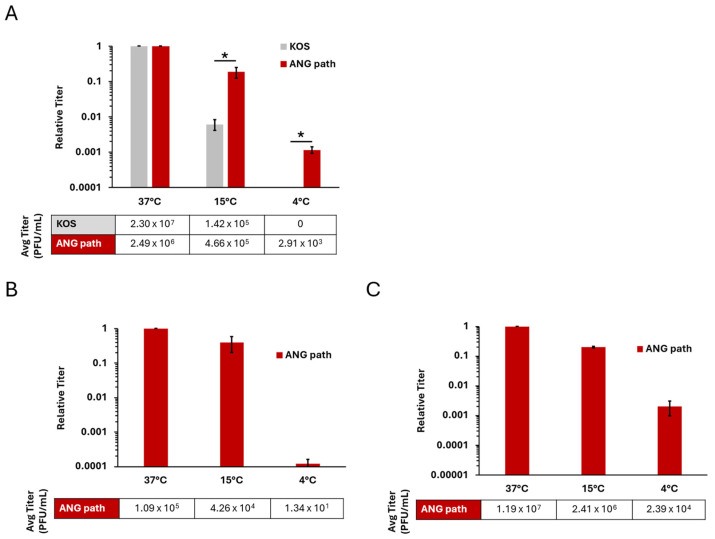
Low temperature entry of HSV-1 ANG path on Vero and CHO cell types. Vero (**A**), CHO-nectin-1 (**B**), and CHO-nectin-2 cells (**C**) were infected with HSV-1 KOS (gray) or HSV-1 ANG path (red) for approximately 80 PFU/well at 37 °C, 15 °C, or 4 °C for 2 h. Cells were then washed with pH 3.0 sodium citrate buffer to inactivate surface-exposed virus, and incubated at 37 °C. At 18–24 hpi, infectivity was measured via plaque assay. Entry at 37 °C was set to 1.0. Results are the mean of triplicate samples from three independent experiments. Error bars represent standard deviation. *, *p* < 0.05, Welch’s *t*-test.

Previous results indicated that HSV-1 strain ANG enters Vero cells at 4 °C, in contrast to the wild type KOS virus which does not enter at 4 °C [33]. The HSV-1 ANG derivative, ANG path entered Vero cells at 4 °C (Figure 1A). There was a ~2 log decrease in ANG path entry at 4 °C relative to 37 °C. In contrast, there was no detectable entry of HSV-1 wild type KOS at 4 °C in Vero cells under these conditions.

HSV-1 ANG path and KOS entered Vero cells at 37 °C and 15 °C. HSV-1 ANG and ANG path can utilize nectin-1 and nectin-2 receptors for entry [38,39]. To address the receptor requirements for low temperature entry, we investigated the ability of ANG path to enter CHO-nectin-1 (Figure 1B) or CHO-nectin-2 cells (Figure 1C). CHO cells are refractory to HSV entry, but expression of a gD-binding receptor renders CHO cells susceptible to HSV-1 entry [41]. HSV-1 ANG path entry at 4 °C was negligible on CHO-nectin-1 cells, suggesting that nectin-1 does not effectively facilitate 4 °C entry (Figure 1B). There was a ~4 log decrease in ANG path entry at 4 °C relative to 37 °C. In contrast, HSV-1 ANG path entered CHO-nectin-2 cells at 4 °C (Figure 1C), at a level similar to that observed in Vero cells (Figure 1A). There was a ~3 log decrease in ANG path entry at 4 °C relative to 37 °C. This indicates that the nectin-2 receptor is a determinant of HSV-1 entry at 4 °C.

### 3.2. HSV-1 ANG Path Enters Human Keratinocytes at 4 °C

**Figure 2:**
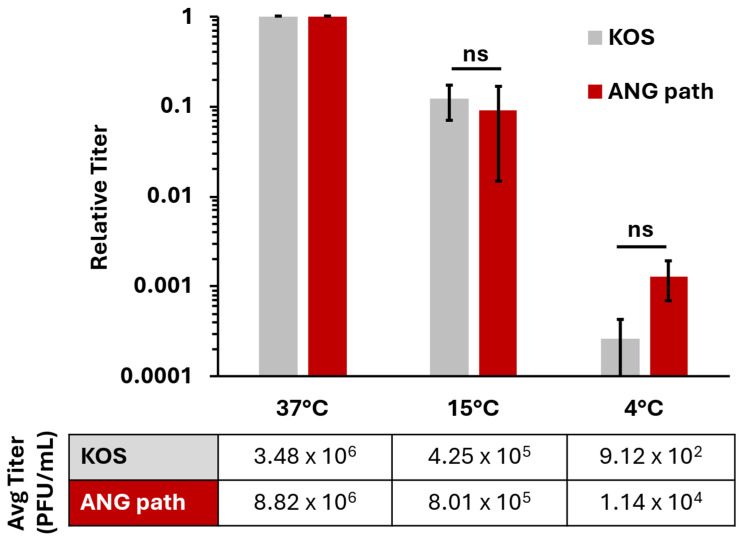
Low temperature entry of HSV-1 on HaCaT cells. Cells were infected with HSV-1 KOS or HSV-1 ANG path for ~80 PFU/well at 37 °C, 15 °C, or 4 °C for 2 h. All cells were then washed with pH 3.0 sodium citrate buffer to inactivate surface exposed virus then incubated at 37 °C. Infectivity was measured via plaque assay at 18–24 hpi. Entry at 37 °C is set to 1.0. Results are the mean of triplicate samples from three independent experiments. Error bars represent standard deviation. ns, not significant, *p* > 0.05, Welch’s *t*-test.

Epithelial cells are the target of primary and recurrent HSV infections. We investigated low-temperature entry of HSV-1 ANG path on a more pathophysiologically relevant cell type, the human epidermal keratinocyte line, HaCaT. In HaCaT cells, wild type HSV-1 entry is facilitated by the nectin-1 receptor [39,42] and occurs via a low pH endocytic pathway [4]. HSV-1 ANG path also enters CHO-nectin-1 cells by a low pH endocytic pathway [15]. Therefore, we hypothesized that HSV-1 ANG path entry into HaCaT cells at 4 °C would be negligible, similar to the results we obtained in CHO-nectin-1 cells. Surprisingly, HSV-1 ANG path entered HaCaT cells at 4 °C, up to 1 log greater than HSV-1 wild type KOS (Figure 2). For both strains, entry was reduced at 4 °C relative to 37 °C; ~3 log for ANG path and ~4 log for KOS. Previous reports indicated that wild type HSV-1 enters keratinocytes at 7 °C [23,37]. Our results suggest that a low level of wild type HSV-1 entry into HaCaT cells persists at 4 °C.

### 3.3. HSV-1 ANG Path Enters HaCaT Cells by a Low pH Endocytic Pathway

**Figure 3:**
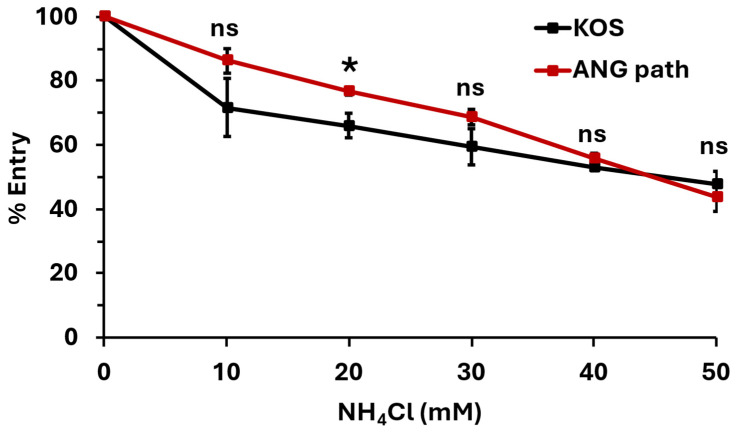
HSV-1 ANG path enters HaCaT cells via a low-pH-dependent pathway. HaCaT cells were treated with ammonium chloride for 1 h at 37 °C. HSV-1 KOS or ANG path was added (100 PFU/well) for 6 h in the continued presence of ammonium chloride. Drug-containing medium was replaced with culture medium. At 18 h post-infection, infectivity was determined by plaque assay. The infectivity of vehicle control (0 mM NH_4_Cl) samples was set to 100%. Results are the mean of quadruplicate samples from three independent experiments. Error bars represent standard deviation. *, *p* < 0.05; ns, *p* > 0.05, Welch’s *t*-test.

We next determined the entry pathway that HSV-1 ANG path utilizes in HaCaT cells. HSV-1 ANG path entry into Vero cells and CHO-nectin-2 cells at 4 °C is robust relative to CHO-nectin-1 cells (Figure 1). This suggests that entry at 4 °C occurs via direct fusion with the plasma membrane. Direct fusion is thought to be a minor entry pathway for wild type HSV-1 in HaCaT cells at 7 °C [23]. Perhaps HSV-1 ANG path is better able to exploit the minor entry pathway of direct penetration in HaCaT cells. Ammonium chloride (NH_4_Cl), a weak base, inhibits HSV-1 entry via the low pH-dependent endocytic pathway by buffering the pH of cellular compartments [1]. We treated HaCaT cells with a range of concentrations of NH_4_Cl and assessed HSV-1 ANG path entry at 37 °C (Figure 3).

Ammonium chloride inhibited entry of both HSV-1 KOS and ANG path in a dose-dependent manner (Figure 3). This suggests that both strains are entering HaCaT cells via a low-pH-dependent pathway, consistent with pH-dependent endocytosis. The effect of ammonium chloride on entry into HaCaT cells at 4 °C was not able to be determined. The combination of ammonium chloride, citrate, and low temperature treatments was lethal to HaCaT cells. Altogether, the results suggest that 4 °C entry of HSV-1 can occur in cell types that support either pH-dependent endocytic (Figure 2 and Figure 3) or direct plasma membrane fusion pathways (Figure 1).

### 3.4. gB Is a Determinant of 4 °C Entry of HSV-1 ANG Path in HaCaT Cells

**Figure 4:**
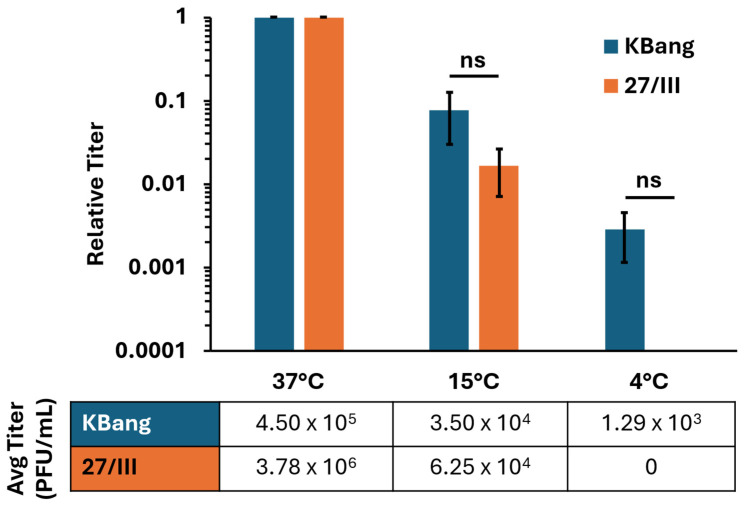
ANG path gB is a determinant of HSV-1 entry into HaCaT cells at 4 °C. HaCaT cells were infected with HSV-1 strains 27/III or KBang (~80 PFU/well) at 37 °C, 15 °C, or 4 °C for 2 h. Cultures were treated with pH 3.0 sodium citrate to inactivate surface-exposed virus. At 18 hpi, infectivity was measured via plaque assay. Results are the means of triplicate samples from three independent experiments. Error bars represent standard deviation. ns, not significant, *p* > 0.05, Welch’s *t*-test.

Many of the unique entry and fusion properties of HSV-1 ANG path are attributed to mutations in the core fusogen, gB. These phenotypes include syncytia formation, fusion from without (FFWO) [18,35,36], and hyperfusogenicity in a cell–cell fusion assay [33]. As such, we hypothesized that ANG path’s hyperfusogenic gB enables HSV-1 ANG path to overcome the energy restrictions of low temperature and contribute to 4 °C entry. We used chimeric HSV-1 strains 27/III and KBang to determine whether ANG path gB contributes to low-temperature entry in HaCaT cells (Figure 4).

HSV-1 27/III is a chimeric ANG path strain in which KOS gB replaces the native gB [35]. 27/III has ANG path gD and is expected to use nectin-2 as a receptor more efficiently than nectin-1. HSV-1 KBang has a KOS background with ANG path gB in place of the native gB [36]. KBang has KOS gD and is expected to use nectin-1 more efficiently than nectin-2, similar to the wild-type virus. HSV-1 KBang exhibited robust entry into HaCaT cells at 4 °C (Figure 4), similar to HSV-1 ANG path (Figure 2). There was a ~3 log decrease in KBang entry at 4 °C relative to 37 °C. In contrast, HSV-1 27/III entry into HaCaT cells was not detectable at 4 °C. This suggests that the hyperfusogenic ANG path gB is responsible for 4 °C entry into HaCaT cells. This contrasts with a previous finding that ruled out a role for ANG path gB in 4 °C entry [43].

## 4. Discussion

**Figure 5:**
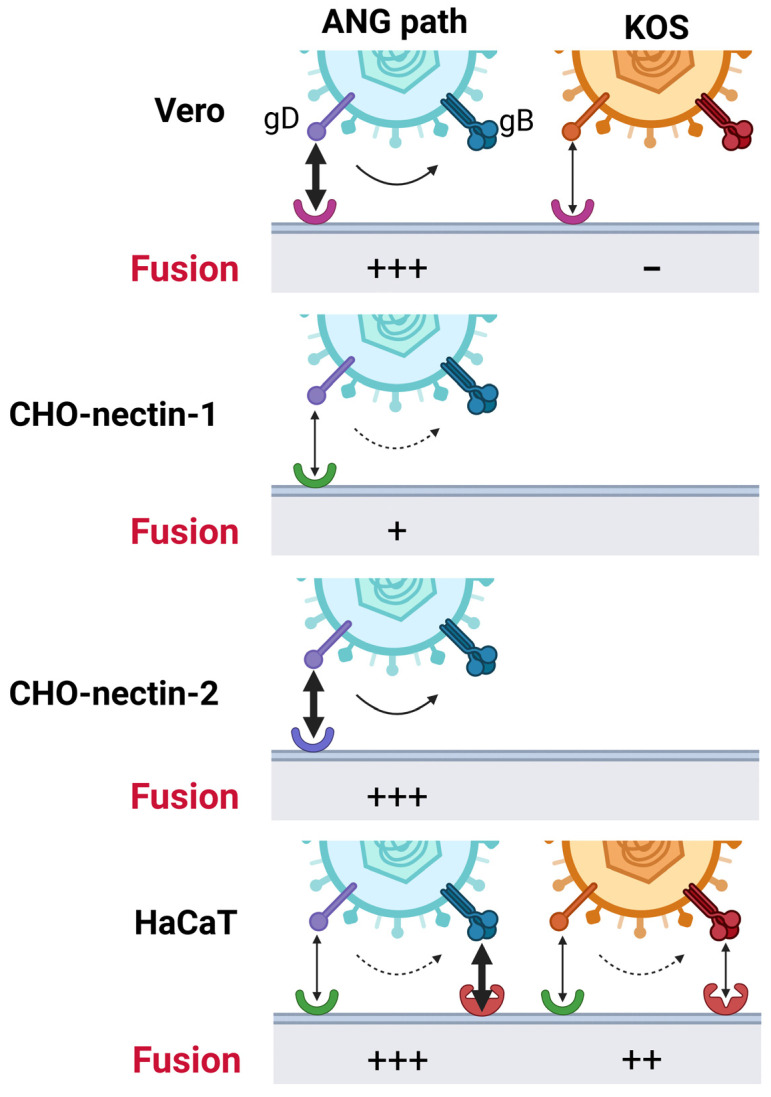
Model of low temperature entry of HSV-1. gD-receptor interactions act as strong (soild-line arrow) or weak (dotted-line arrow) triggers for fusion. In HaCaT cells, additional factors interacting with gB (red receptor) may also activate gB and facilitate 4 °C entry. Receptors: Vero receptor (pink), nectin-1 (green), nectin-2 (blue).

In this study, we demonstrate that nectin-2 and glycoprotein B are protein factors that allow HSV-1 to enter cells at 4 °C. The influence of either factor on 4 °C entry is cell type-dependent, suggesting multiple mechanisms at work (Figure 5). Host cell entry is an essential step in the herpesvirus replicative cycle and is a target for the development of preventative measures and treatments. Low-temperature entry is a rare phenotype for enveloped viruses and has only been described for HSV-1 [23,33,37,43] and Sindbis virus [44]. Low temperatures are inhibitory to HIV cell–cell fusion and influenza virus–cell fusion [30,45] and inactivate respiratory syncytial virus [46]. Studying the atypical phenotypes of HSV-1 ANG path, such as low-temperature entry, provides valuable insight into the mechanisms of HSV-1 fusion and entry.

The results here, together with previous studies, suggest that the proper gD-receptor interaction is one determining factor for 4 °C entry of HSV-1. ANG path gD was previously suggested to be a determinant of 4 °C entry [43]. Nectins are immunoglobulin-like adhesion molecules [47]. Nectin-1 binds to HSV-1 gD to initiate the membrane fusion cascade [48]. Nectin-2 is a receptor for HSV-1 ANG path, some clinical isolates of HSV-1 and HSV-2, and a weak receptor for laboratory strains of HSV-2 [38,49]. HSV-1 strains such as ANG path that carry mutations in gD, e.g., L25, Q27, or T230, permit entry and fusion mediated by nectin-2 [31,34,38,50,51,52]. Nectin-1 is a weak receptor for HSV-1 ANG path [15]. Nectin-2 but not nectin-1 mediates 4 °C entry of HSV-1 ANG path into CHO cells (Figure 1). However, nectin-1 was transiently expressed in CHO-K1 cells in this study, and receptor density was not quantitated, so it is possible that greater 4 °C entry of HSV would result from increased nectin-1 expression. Wild-type soluble gD inhibits entry of wild-type HSV-1 but not HSV-1 ANG path [43,53]. Interestingly, soluble ANG gD has an enhanced inhibitory effect on wild-type HSV-1 entry but fails to inhibit HSV-1 ANG path [43,53]. These results may be explained by differences in receptor affinity and this may also influence low temperature entry. Mutations in ANG path gD possibly affect receptor-triggered conformational change in gD, resulting in enhanced activation of gB fusion function (Figure 5).

ANG path gB is a determinant of 4 °C entry by HSV-1 ANG path in HaCaT cells (Figure 4). ANG path gB has a cytoplasmic tail mutation (A855V) and an ectodomain (V553A) mutation, which together are responsible for hyperfusogenic activity in glycoprotein-mediated cell–cell fusion and for fusion from without (FFWO) [35,36,54,55]. FFWO is virion-mediated cell–cell fusion without de novo viral protein synthesis and is another atypical property of HSV-1 ANG path [56]. FFWO shares key aspects with virus-cell fusion during entry such as dependence on an appropriate gD receptor for fusion. Similarly to 4 °C entry of HSV-1 ANG path, FFWO is only observed when the virus contains ANG path gB and the targets cells express an appropriate receptor [15,18]. In contrast, FFWO does not occur at low temperatures [56], suggesting it is not entirely analogous to 4 °C entry. Low pH-triggered conformational changes in gB mediate HSV-1 fusion during entry into human epithelial cells [57,58]. Interestingly, similar conformational changes occur in ANG path gB [59] and the antigenic conformation of ANG path gB is similar to that of low-pH-treated wild-type gB [18,57], supporting the notion that low pH triggers gB fusion activity and suggesting similarities in the functions of ANG path and KOS gB including similar responses to changes in pH.

We speculate that HSV-1 27/III does not enter HaCaT cells at 4 °C due to the weaker interaction of ANG path gD with the nectin-1 receptor and lack of a hyperfusogenic gB. Conversely, HSV-1 KBang with its nectin-1-binding gD and hyperfusogenic gB exhibits robust 4 °C entry in HaCaT cells (Figure 4). It is not known whether HaCaT cells express nectin-2; thus, the proportion of nectin-1 to nectin-2 in HaCaT cells is also not known. Altogether, low-temperature entry in HaCaT cells may require the contribution of multiple factors such as a specific gD-receptor interaction and hyperfusogenic ANG path gB function. HaCaT cells support 4 °C entry of ANG path, but CHO-nectin-1 cells do so to a lesser extent. Thus, we also propose that HSV-1 gB may function with an unidentified HaCaT cell co-factor to mediate low temperature entry (Figure 5). gB-binding receptors, such as paired immunoglobulin-like type 2 receptor and non-muscle myosin IIA, are potential candidates [16,60,61].

During HSV-1 entry, fusion can occur either directly at the cell plasma membrane (direct penetration) or with an endosomal membrane under the influence of low pH. Selection of entry pathway is determined by a complex combination of virus and cell factors [9,15,16,17,18,19,20,40]. Wild-type HSV-1 enters CHO-receptor cells by endocytosis. HSV-1 ANG path enters CHO-nectin-1 cells via endocytosis but CHO-nectin-2 cells by direct penetration [15]. Also, 4 °C entry of HSV-1 occurs in cell types that favor direct penetration (Vero [3] or CHO-nectin-2) [15] (Figure 1) or the endocytic pathway (HaCaT cells) [4] (Figure 2 and Figure 3). Host cell endocytosis and intracellular trafficking are delayed or inhibited at low temperatures [21,22,23,24] as is viral fusion with host membranes [62,63]. At 37 °C, HSV-1 KOS and ANG path enter HaCaT cells via low-pH-dependent endocytosis. Evidence suggests that HSV-1 entry at 7 °C proceeds primarily by an endocytic mechanism, with a minor role for direct penetration [23]. It remains possible that at 4 °C HSV-1 ANG path enters human keratinocytes via direct fusion at the plasma membrane and does so more efficiently than the wild-type virus. Further investigation is required to determine the precise entry pathway of HSV-1 in HaCaT cells at 4 °C.

Incorporation of the pH 3.0 citrate treatment at 2 hpi in this study allows for isolation and specific investigation of HSV-1 entry, separate from later steps in the replicative cycle. However, it cannot be ruled out that the effects of low temperature on cells persist, impeding HSV-1 replication. Further, these effects may differ across cell types. Low-temperature treatment of cells, including Vero cells, can induce microtubule depolymerization, although normal microtubule structure and distribution is recovered within 4–5 h after cells are returned to 37 °C [64,65]. Following HSV-1 entry into all cells, the nucleocapsid engages dynein/dynactin to travel on microtubules to the nuclear periphery. We do not expect microtubules to impact the fusion reaction at the plasma membrane. Thus, we do not anticipate our measurement of plasma membrane entry to be affected by microtubules. Similarly, in HaCaT cells, the actin cytoskeleton disassembles after incubation at 7 °C, but there is complete recovery after a subsequent 2 h incubation at 37 °C [23]. No change in microtubule morphology or tubulin concentration is detected after cold treatment of CHO-K1 cells [66]. In this study, following low-temperature incubation and citrate treatment, all cells are incubated at 37 °C for a minimum of 16 h, allowing for microtubule assembly. While steps downstream of HSV-1 entry may initially be delayed, we do not expect a significant effect on plaque formation measured at >16 h.

Overall, our findings support selective roles for both the gD–receptor interaction and gB in low-temperature entry of HSV-1. Further investigation of the unique entry and fusion phenotypes of HSV-1 ANG path and determinants of low temperature entry across cell types will contribute to our understanding of the complex determinants of HSV-1 entry.

## Data Availability

The raw data supporting the conclusions of this article will be made available by the authors on request.

## Abbreviations

The following abbreviations are used in this manuscript:

HSV-1	Herpes simplex virus 1
FFWO	Fusion-from-without
gB	Glycoprotein B
gD	Glycoprotein D
